# Author Correction: The molecular basis of force selectivity by PIEZO2

**DOI:** 10.1038/s41586-026-11079-1

**Published:** 2026-09-10

**Authors:** Eric M. Mulhall, Oleg Yarishkin, Rose Z. Hill, Anna K. Koster, Ardem Patapoutian

**Affiliations:** 1 Howard Hughes Medical Institute, Department of Neuroscience, Dorris Neuroscience Center, Scripps Research, La Jolla, CA USA; 2 Vollum Institute, Oregon Health & Science University, Portland, OR USA; 3 Present Address: Department of Chemical Physiology and Biochemistry, Oregon Health & Science University, Portland, OR USA

**Keywords:** Single-molecule biophysics, Ion channels, Super-resolution microscopy, Ion channels in the nervous system

Correction to: *Nature* https://doi.org/10.1038/s41586-026-10182-7 Published online 4 March 2026

In the version of this article initially published, in the Fig. 4d Source Data and associated figure plot, due to an error during the full width at half maximum resolution analysis using the ‘fwhm_on_spots’ jython-fiji macro, one channel from one image was inadvertently loaded twice into the analysis script. The corrected analysis changes the PIEZO2 FWHM from 83 ± 31 nm to 88 ± 32 nm and the number of PIEZO2 puncta from *n* = 201 to *n* = 320. The figure panel and Source Data are now updated. In the Fig. 4d legend, the reported *n* value for FLNB puncta was incorrect and is now updated to *n* = 468 rather than 469.

In the Source Data for Figs. 1–3, the “Raw MINFLUX output” tabs for cell #2 of the “PIEZO2 TCOK105 – Isotonic” condition contained values from an analysis version that used a different standard-deviation-per-trace threshold from that used for the figures. Separately, in the “Raw MINFLUX output” tab in the Source Data of Fig. 1 and Extended Data Fig. 4 for cell #3 of the “mPIEZO1 TCO*K103 – Hypotonic” condition, an analysis output was uploaded in which two molecules that should have been excluded by the *z*-axis filtering criterion were inadvertently retained. (The Source Data Extended Data Fig. 4 file was originally mislabeled Source Data Extended Data Fig. 3).

Following the Source Data updates, the Fig. 1b legend now reports *n* = 102 instead of *n* = 52 molecules for the unstimulated PIEZO2 condition; Fig. 1d now reports a median of 37.1 nm and *n* = 39 molecules instead of 34.7 nm and *n* = 41 molecules; Fig. 1e now reports 20.8 nm instead of 19.9 nm for the unstimulated PIEZO2 condition; the PIEZO2 values in Fig. 3e now report median = 20.8 nm and *n* = 102 molecules rather than median = 18.0 nm and *n* = 30 molecules. In the Fig. 1–3 legends, in three cases, mean values were reported rather than the correct median values, where for Fig. 1h, PIEZO1 hyperosmotic condition, 19.8 nm has been updated to 17.1 nm; for Fig. 2b, PIEZO2 + cytochalasin D at rest, 20.1 nm has been updated to 18.0 nm; for Fig. 3e, *Flnb* DsiRNA + hypo-osmotic swelling, 37.0 nm has been updated to 38.7 nm. In the Fig. 1 and Extended Data Fig. 2 legends, sample sizes were listed incorrectly, where for Fig. 1k, PtK2 control cells, *n* = 6 has been updated to *n* = 7 cells; for Extended Data Fig. 2d, direct Alexa 647, *n* = 2,014 has been updated to 2,013 traces, fluorogenic DNA PAINT, *n* = 7,254 has been updated to 7,253 traces, and for traditional DNA PAINT, *n* = 1,013 has been updated to 1,012 traces. In the Extended Data Fig. 8c legend, a typographical error in “Piezo2 *Flnb* KO” has been corrected to “Piezo1 *Flnb* KO”.

The corrections do not affect the outcome or significance of any reported statistical comparison, the direction of any experimental result, or the scientific conclusions or interpretations presented in the article. Figure 4d, the legends for Figs. 1–4 and Extended Data Figs. 2 and 8, and Source Data for Figs. 1–4 and Extended Data Fig. 4 are now updated in the HTML and PDF versions of the article. For comparison, the original Fig. 4 is available as Supplementary Information accompanying this amendment.

**Supplementary information** is available in the online version of this amendment.

## Supplementary information

Original Fig. 4

