# Supplementary material for: The molecular basis of force selectivity by PIEZO2
Source: Nature. 2026 Mar 4;653(8113):297–305. doi: 10.1038/s41586-026-10182-7 (PMC13149025; doi:10.1038/s41586-026-10182-7)
Supplement: Supplementary file 1 — Supplementary Notes 1 and 2 and Supplementary Tables 1–3. [file 41586_2026_10182_MOESM1_ESM.pdf]

nature portfolio

https://doi.org/10.1038/s41586-026-10182-7

Supplementary information

The molecular basis of force selectivity by PIEZO2

In the format provided by the
authors and unedited

Supplementary Notes

Supplementary Note 1 | Sequences of CRISPR sgRNAs, hFlnb genotyping primers, dsiRNAs, and fluorogenic DNA PAINT docking and imaging strands.

Sequences of CRISPR sgRNAs (5'-3'):

sgRNA 1	UGUGGAGGUGGAAGAUCCCC
sgRNA 2	UGUACACACAUCGAUACACC
sgRNA 3	GUCCCUUCGUUGUGCAGGUU

h*Flnb* genotyping primers (5'-3'):

forward	TCAACCATAAACCCTTCAACCC
reverse	GAGAAAAATGCGTACCCCCAG
sequencing	ACACTGGTCCTGTTTGCATTTTG

Target sequences of *H. Sapien* ON-TARGETplus SMARTpool siRNAs (5'-3'):

CTNNB1	GAUCCUAGCUAUCGUUCUU
	UAAUGAGGACCUAUACUUA
	GCGUUUGGCUGAACCAUCA
	GGUACGAGCUGCUAUGUUC
TAGLN2	GCUCAUUAAUGCACUGUAC
	GGCAGUAGCCCGAGAUGAU
	GCAAGAACGUGAUCGGGUU
	GAACAUGGCCUGUGUGCAG
FLNB	GCGAUGCAGUGAAGGAUUU
	GCACGGUCACUGUUAGAUA
	CAAGGUAGCCAUCCUCAGA
	UACAUUCGAUGACCAUAAA
MSN	CGUAUGCUGUCCAGUCUAA
	GAGGGAAGUUUGGUUCUUU
	UCGCAAGCCUGAUACCAUU
	GGCUGAAACUCAAUAAGAA
FLNA	GCAGGAGGCUGGCGAGUAU
	GCACCCAGACCGUCAAUUA
	GCACAUGUUCCGUGUCCUA
	GAAUGGCGUUUACCUGAUU
VCL	UGAGAUAAUUCGUGUGUUA
	GAGCGAAUCCCAACCAUAA
	GCCAAGCAGUGCACAGAUA
	CAGCAUUUAUUAAGGUUGA

Non-targeting	UGGUUUACAUGUCGACUAA<br>UGGUUUACAUGUUGUGUGA<br>UGGUUUACAUGUUUUCUGA<br>UGGUUUACAUGUUUUCCUA

P. tridactylis Flnb dsiRNAs (5’-3’):

dsiRNA 1	rGrArArUrUrCrArCrArArUrArGrArUrArCrCrArArArGrGAG<br>rCrUrCrCrUrUrUrGrGrUrArUrCrUrArUrUrGrUrGrArArUrUrCrArG
dsiRNA 2	rCrCrArUrCrGrArUrArGrCrArArArGrCrUrArUrUrGrUrUGA<br>rUrCrArArCrArArUrArGrCrUrUrUrGrCrUrArUrCrGrArUrGrGrArC
dsiRNA 3	rArGrUrGrArUrArArGrArArCrArArGrArCrUrUrArUrUrCTG<br>rCrArGrArArUrArArGrUrCrUrUrGrUrUrCrUrUrArUrCrArCrUrGrU
dsiRNA 4	rGrCrArUrUrCrArCrArCrUrArUrUrGrArUrGrUrCrArArGTT<br>rArArCrUrUrGrArCrArUrCrArArUrArGrUrGrUrGrArArUrGrCrCrA

M. musculus Flnb dsiRNAs (5’-3’):

dsiRNA 1	rGrGrUrArUrCrCrArArUrCrArGrArArUrUrCrUrUrCrArUCA<br>rUrGrArUrGrArArGrArArUrUrCrUrGrArUrUrGrGrArUrArCrCrUrG
dsiRNA 2	rGrGrCrGrUrUrUrArCrCrArArCrArArArUrCrUrArArUrGTC<br>rGrArCrArUrUrArGrArUrUrUrGrUrUrGrGrUrArArArCrGrCrCrUrC
dsiRNA 3	rGrUrCrUrArCrUrCrArArGrCrGrArUrArArCrCrArArArCAA<br>rUrUrGrUrUrUrGrGrUrUrArUrCrGrCrUrUrGrArGrUrArGrArCrCrA
dsiRNA 4	rArCrUrGrGrArUrCrUrGrArGrCrArArGrArUrArArArArATT<br>rArArUrUrUrUrUrArUrCrUrUrGrCrUrCrArGrArUrCrCrArGrUrGrG

Fluorogenic DNA PAINT docking and imaging strands:

Docking strand 5' - CCTCGCTGAACCTCTTA/Tetrazine - 3'
Imaging strand 5' - ATTO643/AAGAAGTAAAGGGAG/IowaBlackFQ - 3'

Supplementary Note 2 | Pseudocode for MINFLUX analysis

A) PIEZO trimer clustering from 3D MINFLUX localizations

Inputs
• An exported .mat file from Abberior Imspector software, containing localization and metadata arrays, including itr.loc, itr.lnc, itr.efo, itr.fbg, itr.ecc, itr.eco, itr.cfr, and a trace identifier vector tid.
• User-specified parameters: stdev_trace_threshold (per-trace error), loc_per_trace_threshold (minimum localizations per trace), efo_cutoff (photon emission frequency), optional spatial windows (X, Y, and/or Z), DBSCAN/GMM parameters (db1_size, db1_minPts, db2_size, db2_minPts, gmm_sigma), nearest-neighbor limits (minDist, neighborDist), angle_threshold.

Outputs
• Results table with columns: X, Y, Z, stdev X, stdev Y, stdev Z, DBSCAN ID, Inter-blade angle, Inter-

blade distance, Average inter-blade distance.
• Analyzed .mat file containing Results and intermediate arrays.

Procedure

1. Load data.
   Extract per-localization coordinates from the last MINFLUX iteration:
   x = itr.loc(:,10,1), y = itr.loc(:,10,2), z = 0.7 × itr.loc(:,10,3) (meters; 0.7 corrects refractive index mismatch).
   Extract quality metrics: efo = itr.efo(:,10), fbg = itr.fbg(:,10).
   Convert tid to double (column vector).
2. Assemble per-localization records.
   traces = [x, y, z, tid, efo, fbg] (columns 1–6).
3. Apply filters.
   3.1 Localizations per trace: compute counts per tid; retain rows whose tid count exceeds loc_per_trace_threshold.
   3.2 EFO gate: remove rows with efo > efo_cutoff.
   3.3 Per-trace spatial stability: for each tid, compute σx, σy, σz; drop the entire trace if any σ ≥ stdev_trace_threshold.
   3.4 (Optional) Apply spatial windows in Z (and/or X/Y) when empirically justified.
4. Unit normalization for downstream algorithms.
   For visualization, traces_nm = traces × $1e^9$ (nm). For clustering, take filt_XYZ_m = traces(:,1:3) in meters, then provide {filt_XYZ_m × $1e^6$} (µm) to the clustering routine to match its expected units.
5. Two-stage density clustering with GMM refinement.
   Use algorithm "dbscan2" (originally described in Pape, et al.) with parameters db1_size, db1_minPts, db2_size, db2_minPts, and gmm_sigma.
   Obtain per-cluster centers clust_centers (K×3) and per-axis standard deviations clust_stdevs (K×3); both are in nanometers.
6. Exclude unreliable GMM fits.
   Retain clusters whose all three per-axis standard deviations are < error_threshold_nm and non-NaN.
7. Identify trimer candidates by DBSCAN on cluster centers.
   Run DBSCAN with epsilon_nm (e.g., 60 nm) and minpts = 3 on clust_centers.
   Remove noise points (label = −1).
   Keep only groups whose label occurs exactly three times (putative trimers).
   Create clust_DBSCAN_3 = [X, Y, Z, stdevX, stdevY, stdevZ, label] and sort by label.
8. Independent nearest-neighbour (NN) check.
   Compute pairwise Euclidean distances among retained centers.
   Define valid neighbours by minDist_nm ≤ distance ≤ neighborDist_nm (e.g., 6–60 nm).
   For each point, require exactly two neighbours in this interval.
   Keep only points passing the NN criterion and carry forward as clust_NN.
9. Intersection of DBSCAN and NN selections.
   Match cluster identities between clust_DBSCAN_3 and clust_NN.
   Retain labels present in both sets and verify exactly three rows per label.
   Order final rows as [X, Y, Z, stdevX, stdevY, stdevZ, label].
10. Inter-blade angles.
    For each label (trimer), let P = {p1, p2, p3} be the three XYZ points (nm).
    For j = 1..3, define v1 = p(next(j)) − p(j), v2 = p(prev(j)) − p(j).
    Angle(j) = arccos(dot(v1/‖v1‖, v2/‖v2‖)) in degrees.
    Append the per-point angle as a new column.

11. Optional angular filtering.
    If angle_threshold < x°, discard any trimer for which any of its three angles exceeds angle_threshold. Default is off.
12. Inter-blade distances.
    For each trimer, compute pairwise distances d_1-2, d_2-3, d_3-1.
    Assign a representative edge distance per row (column 9).
    Compute the average inter-blade distance $\bar{d}$ = mean(d_1-2, d_2-3, d_3-1) and write to one representative row of the trimer (column 10), leaving the other two blank.
13. Finalization.
    Assemble Results with the specified columns.
    Save <input>_analyzed.mat containing Results and intermediate arrays.

Computational considerations
• Pairwise distances over K cluster centers scale as $O(K^2)$.
• DBSCAN typically scales between O(K log K) and $O(K^2)$ depending on neighbourhood indexing.
• Angle and distance computations scale linearly with K.

Notes
• Unit consistency: GMM outputs and DBSCAN epsilon operate in nm; raw traces enter in meters.
• The 0.7 refractive index correction factor for z should be applied prior to any thresholding or clustering (if using a standard oil objective).
• The localizations-per-trace filter is strict "> threshold" (not ≥) as in the reference script; adapt if needed for boundary behavior.

B) Single-molecule diffusion analysis from tracked MINFLUX data

Inputs
• folder_path pointing to a directory containing one or more .mat files (subfolder search permitted).
• Each file provides itr.loc(:,5,1:3), itr.efo(:,5), itr.fbg(:,5), tid, and tim.

Outputs
• Per-trajectory microscopic diffusion coefficients (D_micro) and corresponding R^2 values.
• Per-trajectory macroscopic diffusion coefficients (D_macro) and corresponding R^2 values.
• Binned, weighted ensemble microscopic/macroscopic MSD with SEM; fitted D_ensemble.
• Optional <foldername>_analyzed.mat and figures.

Procedure
1. Load and concatenate.
   Recursively enumerate .mat files under folder_path.
   For each file: load x = itr.loc(:,5,1), y = itr.loc(:,5,2), z = itr.loc(:,5,3), time = tim', id = double(tid)', quality metrics efo = itr.efo(:,5), fbg = itr.fbg(:,5).
   Stack into traces = [x, y, 0.7×z, id, efo, fbg, time] (meters; ids; quality; seconds).
2. Quality, continuity, and stability filtering.
   2.1 Remove rows with efo > efo_cutoff.
   2.2 Count localizations per id; retain rows whose id count exceeds loc_per_trace_threshold.
   2.3 For each id, compute σx, σy, σz and their mean; drop the id if mean σ ≥ stdev_trace_threshold.
   2.4 Temporal continuity: for each id, sort by time; compute successive gaps; truncate the trajectory at the first gap exceeding time_gap_threshold; append the retained segment.

3. Convert positions for MSD analysis.
   Move to micrometers: [x, y, z] × $1e^6$; retain id, efo, fbg, time unchanged.
   The working array becomes [xµm, yµm, zµm, id, efo, fbg, time_s].
4. Per-trajectory microscopic MSD and D_micro.
   For each id:
   • Sort by time.
   • Let the trajectory length be N; if N < 2, record NaNs and continue.
   • For lag $\tau = 1..N-1$, compute $\delta^2(\tau) = \text{mean}[(\Delta x)^2 + (\Delta y)^2 + (\Delta z)^2]$, where Δ denotes displacement separated by τ samples. Also compute the mean time lag corresponding to τ, and set the weight as the number of contributing displacement pairs.
   • Remove extreme outliers (e.g., $\delta^2 > 10\ \mu m^2$) prior to fitting.
   • Restrict to lags with minFitDelay_micro ≤ τ_time ≤ maxFitDelay_micro; if fewer than two points remain, set D_micro and R^2 to NaN.
   • Otherwise, fit a straight line to MSD versus τ_time (per-trajectory, unweighted is sufficient here); convert the slope to D_micro via MSD = 6Dτ (3D diffusion). Record R^2.
5. Ensemble microscopic MSD (binned, weighted) and D_ensemble.
   Pool all per-trajectory (τ_time, MSD, weight) triplets across trajectories.
   Bin τ_time into ∼100 contiguous bins over its observed range; within each bin compute the weighted mean MSD = Σ(w·MSD)/Σ(w), the weighted variance, the effective sample size n_eff = $(\Sigma w)^2/\Sigma(w^2)$, and the standard error of the mean (SEM) = √(var_w/n_eff).
   Retain only bins with minFitDelay_micro ≤ τ_time ≤ maxFitDelay_micro.
   Perform a weighted linear regression of MSD versus τ_time using bin weights; report D_ensemble = slope/6 and R^2.
6. Ensemble macroscopic MSD (longer delays) and D_mac.
   From the same binned series, select bins with minFitDelay_macro ≤ τ_time ≤ maxFitDelay_macro.
   Perform a weighted linear regression; report D_mac = slope/6 and R^2.
7. Post-filters and summaries.
   Remove per-trajectory D_micro and D_macro values > 10 $\mu m^2/s$ (non-physical for membrane proteins under typical conditions).
   Apply a per-trajectory fit-quality filter: retain only D_micro and D_macro with $R^2 \geq$ min_r2 for distributions and descriptive summaries.
8. Visualization (optional).
   Plot a boxplot of per-trajectory microscopic R^2.
   Plot ensemble microscopic and macroscopic MSD with SEM and fitted lines annotated by D and R^2.
   Plot normalized trajectory overlays for tracks spanning at least track_overlay_time seconds; center each track by its center of mass (or initial-position normalization).
9. Save (optional).
   Write <foldername>_analyzed.mat containing key variables (per-trajectory MSD lists, ensemble series, D estimates, fit objects, and masks) and figures where appropriate.

<u>Computational considerations</u>
• None

<u>Notes</u>
• Coordinates are in µm for MSD computations; diffusion coefficients are reported in $\mu m^2\ s^{-1}$.
• The refractive-index correction (0.7 on z) is applied prior to filtering.
• The ensemble SEM uses an effective sample size (n_eff) appropriate for weighted means.
• The R^2 threshold (min_r2) controls the stringency with which poorly fit trajectories are excluded from summary statistics.

Supplementary Table 1 | Statistics tables comparing replicates between MINFLUX structural imaging conditions.

A Kruskal-Wallis test with Dunn’s post hoc test was used to assess replicate variability across each condition.

mPIEZO1 TCO*K 103 - No Stimulation

Kruskal-Wallis test	
P value	0.4291
Number of groups	4
Kruskal-Wallis statistic	2.766

Dunn's multiple comparisons test	Adjusted P Value
1 vs. 2	>0.9999
1 vs. 3	0.7557
1 vs. 4	>0.9999
2 vs. 3	>0.9999
2 vs. 4	>0.9999
3 vs. 4	>0.9999

mPIEZO1 TCO*K 103 - Hypotonic

Kruskal-Wallis test	
P value	0.0102
Number of groups	3
Kruskal-Wallis statistic	9.169

Dunn's multiple comparisons test	Adjusted P Value
1 vs. 2	>0.9999
1 vs. 3	0.0441
2 vs. 3	0.011

mPIEZO1 TCO*K 103 - Hypertonic

Kruskal-Wallis test	
P value	0.1561
Number of groups	3
Kruskal-Wallis statistic	3.746

Dunn's multiple comparisons test	Adjusted P Value
1 vs. 2	0.5625
1 vs. 3	>0.9999
2 vs. 3	0.1898

PIEZO2 TCO*K 105 - Isotonic

Kruskal-Wallis test	
P value	0.4291
Number of groups	4
Kruskal-Wallis statistic	2.766

Dunn's multiple comparisons test	Adjusted P Value
1 vs. 2	>0.9999
1 vs. 3	0.7557
1 vs. 4	>0.9999
2 vs. 3	>0.9999
2 vs. 4	>0.9999
3 vs. 4	>0.9999

PIEZO2 TCO*K 105 - Hypotonic

Kruskal-Wallis test	
P value	0.0938
Number of groups	3
Kruskal-Wallis statistic	4.733

Dunn's multiple comparisons test	Adjusted P Value
1 vs. 2	0.1021
1 vs. 3	>0.9999
2 vs. 3	0.5136

PIEZO2 TCO*K 105 - Hypertonic

Kruskal-Wallis test	
P value	0.1613
Number of groups	3
Kruskal-Wallis statistic	3.649

Dunn's multiple comparisons test	Adjusted P Value
1 vs. 2	>0.9999
1 vs. 3	0.7444
2 vs. 3	0.209

PIEZO2 TCO*K 105 - Cytochalasin D - Isotonic

Kruskal-Wallis test	
P value	0.044
Number of groups	3
Kruskal-Wallis statistic	6.245

Dunn's multiple comparisons test	Adjusted P Value
1 vs. 2	>0.9999
1 vs. 3	0.1586
2 vs. 3	0.0562

PIEZO2 TCO*K 105 - Cytochalasin D - Hypotonic

Kruskal-Wallis test	
P value	0.195
Number of groups	5
Kruskal-Wallis statistic	6.057

Dunn's multiple comparisons test	Adjusted P Value
1 vs. 2	>0.9999
1 vs. 3	>0.9999
1 vs. 4	>0.9999
1 vs. 5	>0.9999
2 vs. 3	>0.9999
2 vs. 4	>0.9999
2 vs. 5	>0.9999
3 vs. 4	>0.9999
3 vs. 5	>0.9999
4 vs. 5	>0.9999

PIEZO2 TCO*K 105 - IDR5 Delete - Isotonic

Kruskal-Wallis test	
P value	0.7964
Number of groups	4
Kruskal-Wallis statistic	1.02

Dunn's multiple comparisons test	Mean rank diff.
1 vs. 2	-2.856
1 vs. 3	-1.939
1 vs. 4	-7.273
2 vs. 3	0.9167
2 vs. 4	-4.417
3 vs. 4	-5.333

PIEZO2 TCO*K 105 - IDR5 Delete - Hypotonic

Kruskal-Wallis test	
P value	0.7768
Number of groups	6
Kruskal-Wallis statistic	2.498

Dunn's multiple comparisons test	Adjusted P Value
1 vs. 2	>0.9999
1 vs. 3	>0.9999
1 vs. 4	>0.9999
1 vs. 5	>0.9999
1 vs. 6	>0.9999
2 vs. 3	>0.9999
2 vs. 4	>0.9999
2 vs. 5	>0.9999
2 vs. 6	>0.9999
3 vs. 4	>0.9999
3 vs. 5	>0.9999
3 vs. 6	>0.9999
4 vs. 5	>0.9999
4 vs. 6	>0.9999
5 vs. 6	>0.9999

PIEZO2 TCO*K 105 - FLNB dsiRNA - Isotonic

Kruskal-Wallis test	
P value	0.7472
Number of groups	3
Kruskal-Wallis statistic	0.5828

Dunn's multiple comparisons test	Adjusted P Value
1 vs. 2	>0.9999
1 vs. 3	>0.9999
2 vs. 3	>0.9999

PIEZO2 TCO*K 105 - FLNB dsiRNA - Hypotonic

Kruskal-Wallis test	
P value	0.2639
Number of groups	3
Kruskal-Wallis statistic	2.665

Dunn's multiple comparisons test	Adjusted P Value
1 vs. 2	>0.9999
1 vs. 3	>0.9999
2 vs. 3	0.332

mPIEZO1 TCO*K 103 - Cytochalasin D - Isotonic

Kruskal-Wallis test	
P value	0.8145
Number of groups	3
Kruskal-Wallis statistic	0.4103

Dunn's multiple comparisons test	Adjusted P Value
1 vs. 2	>0.9999
1 vs. 3	>0.9999
2 vs. 3	>0.9999

mPIEZO1 TCO*K 103 - Cytochalasin D - Hypotonic

Kruskal-Wallis test	
P value	0.5818
Number of groups	3
Kruskal-Wallis statistic	1.083

Dunn's multiple comparisons test	Adjusted P Value
1 vs. 2	0.9588
1 vs. 3	>0.9999
2 vs. 3	>0.9999

Supplementary Table 2 | MINFLUX 3D imaging sequence parameters

Iteration	Pattern	patGeoFactor	phtLimit	patDwellTime	patRepeat	pwrFactor	ccrLimit	bgcThreshold	stickiness
0	hexagon	0.8	160	0.001	1	1	-1	15000	-
1	zline	0.8, 4.0	400	0.001	1	1	-1	15000	2
2	square	0.8	100	0.001	5	1	0.9	10000	2
3	zline2	0.8	50	0.001	5	1	-1	10000	2
4	square	0.42	67	0.001	5	2	-1	10000	2
5	zline2	0.42	33	0.001	5	2	-1	10000	2
6	square	0.21	67	0.001	5	4	0.8	10000	2
7	zline2	0.21	33	0.001	5	4	-1	10000	2
8	square	0.11	100	0.001	5	6	-1	10000	2
9	zline2	0.11	50	0.001	5	6	-1	10000	2

Supplementary Table 3 | MINFLUX 3D tracking sequence parameters

Iteration	Pattern	patGeoFactor	phtLimit	patDwellTime	patRepeat	pwrFactor	ccrLimit	bgcThreshold	stickiness
0	hexagon	0.8	40	0.0005	1	1	-1	30000	-
1	zline	0.8, 4.0	300	0.002	1	1	-1	30000	4
2	octahedron	0.8	40	0.0002	1	1	-1	35000	4
3	octahedron	0.42	30	0.0002	1	2	0.9	40000	4
4	octahedron	0.28	30	0.0002	1	3	-1	60000	4
