# Supplementary material for: The molecular basis of force selectivity by PIEZO2
Source: Nature. 2026 Mar 4;653(8113):297–305. doi: 10.1038/s41586-026-10182-7 (PMC13149025; doi:10.1038/s41586-026-10182-7)
Supplement: Supplementary file 2 — Reporting Summary [file 41586_2026_10182_MOESM2_ESM.pdf]

nature portfolio

Corresponding author(s): Ardem Patapoutian
Eric Mulhall

Last updated by author(s): Jan 15, 2025

Reporting Summary

Nature Portfolio wishes to improve the reproducibility of the work that we publish. This form provides structure for consistency and transparency in reporting. For further information on Nature Portfolio policies, see our Editorial Policies and the Editorial Policy Checklist.

Statistics

For all statistical analyses, confirm that the following items are present in the figure legend, table legend, main text, or Methods section.

n/a	Confirmed	
☐	☒	The exact sample size (*n*) for each experimental group/condition, given as a discrete number and unit of measurement
☐	☒	A statement on whether measurements were taken from distinct samples or whether the same sample was measured repeatedly
☐	☒	The statistical test(s) used AND whether they are one- or two-sided<br>*Only common tests should be described solely by name; describe more complex techniques in the Methods section.*
☒	☐	A description of all covariates tested
☐	☒	A description of any assumptions or corrections, such as tests of normality and adjustment for multiple comparisons
☐	☒	A full description of the statistical parameters including central tendency (e.g. means) or other basic estimates (e.g. regression coefficient) AND variation (e.g. standard deviation) or associated estimates of uncertainty (e.g. confidence intervals)
☐	☒	For null hypothesis testing, the test statistic (e.g. *F*, *t*, *r*) with confidence intervals, effect sizes, degrees of freedom and *P* value noted<br>*Give P values as exact values whenever suitable.*
☒	☐	For Bayesian analysis, information on the choice of priors and Markov chain Monte Carlo settings
☒	☐	For hierarchical and complex designs, identification of the appropriate level for tests and full reporting of outcomes
☐	☒	Estimates of effect sizes (e.g. Cohen's *d*, Pearson's *r*), indicating how they were calculated

Our web collection on statistics for biologists contains articles on many of the points above.

Software and code

Policy information about availability of computer code

Data collection	MINFLUX data were acquired on a commercial MINFLUX 3D microscope an Olympus IX83 microscope body (Abberior Instruments) using Imspector software (v16.3.15645-m2205) with MINFLUX drivers. Confocal images were collected with NIS-Elements software (version 5.40.01, Nikon). STED imaging was performed on an Abberior Instruments Facility Line 3D STED microscope on an Olympus IX83 microscope body using Lightbox 2025 software (version: 2024.48.21878-gc86bbd647c). Electrophysiology data was collected with pClamp software (version 10.2 and 10.7, Molecular Devices).
Data analysis	MINFLUX data analysis was performed using custom code written in MATLAB (version R2021b, MathWorks). Code for structure data and tracking analysis is available at https://github.com/PatapoutianLab/MINFLUX_Localization_and_Tracking_Analysis (https://doi.org/10.5281/zenodo.17625937). Confocal images were analyzed using Fiji (version 2.16.0/1.54p, https://fiji.sc/). STED data was analyzed and deconvolved using Lightbox 2025 software (version: 2024.48.21878-gc86bbd647c). STED colocalization was performed in Fiji using the Coloc 2 plugin (version 3.1.0). FWHM calculations were performed with fwhm_on_spots (https://github.com/sommerc/spots_fwhm). DNA sequences were created and analyzed in SnapGene (Version 8.0.3, Dotmatics). Data visualization and statistical tests were performed with MATLAB 2023 (version R2023b, MathWorks) and Prism (version 10.4.2, GraphPad). Visualization of localizations in Extended Data Fig. 2a were performed using ParaView (version 5.10, kitware). Molecular structures were visualized using MolStar viewer (https://molstar.org/viewer/, version 5.4.1) and Chimera software (version 1.15, UCSF). Graphics were created using Adobe Illustrator (version 2025, Adobe) and BioRender (biorender.com). Sanger sequencing traces of FLNB KO cells were analyzed with the Editco ICE analysis tool (https://ice.editco.bio/#/). Peptides identified from mass spectometry were analyzed using Mascot (Matrix Science, version 2.8.0).

For manuscripts utilizing custom algorithms or software that are central to the research but not yet described in published literature, software must be made available to editors and reviewers. We strongly encourage code deposition in a community repository (e.g. GitHub). See the Nature Portfolio guidelines for submitting code & software for further information.

Data

Policy information about availability of data

All manuscripts must include a data availability statement. This statement should provide the following information, where applicable:

- Accession codes, unique identifiers, or web links for publicly available datasets
- A description of any restrictions on data availability
- For clinical datasets or third party data, please ensure that the statement adheres to our policy

Data supporting the article, including the raw MINFLUX analysis output for each experimental condition, are provided as source data. Published protein structures were obtained from the RSCB Protein Data Bank (PIEZO1 6B3R and PIEZO2 6KG7). AlphaFold III models were generated with the Google DeepMind AlphaFold Server. Raw data is available at: https://doi.org/10.5281/zenodo.17644763. All reagents that are not commercially available are available from the corresponding authors upon reasonable request. Source data are provided with this paper. For mass spectometry proteomics, all samples were analyzed using Mascot software (Matrix Science) with human proteins contained in the NCBInr protein database, assuming the digestion enzyme trypsin. Hits were cross referenced against human proteins in the UniprotKB human proteome (UP000005640).

Research involving human participants, their data, or biological material

Policy information about studies with human participants or human data. See also policy information about sex, gender (identity/presentation), and sexual orientation and race, ethnicity and racism.

Reporting on sex and gender	N/A
Reporting on race, ethnicity, or other socially relevant groupings	N/A
Population characteristics	N/A
Recruitment	N/A
Ethics oversight	N/A

Note that full information on the approval of the study protocol must also be provided in the manuscript.

Field-specific reporting

Please select the one below that is the best fit for your research. If you are not sure, read the appropriate sections before making your selection.

☒ Life sciences ☐ Behavioural & social sciences ☐ Ecological, evolutionary & environmental sciences

For a reference copy of the document with all sections, see nature.com/documents/nr-reporting-summary-flat.pdf

Life sciences study design

All studies must disclose on these points even when the disclosure is negative.

Sample size	No analyses were performed in advance to predetermine sample size. Samples sizes were selected based upon prior studies in the literature using comparable assays and effect sizes (e.g. Mulhall, et al., 2023, Ranade, et al., 2014) and on feasibility constraints of MINFLUX imaging and patch clamp recording. For each experiment type, we aimed to include at least three independent biological replicates (independent cultures/ animals prepared on different days) and to repeat experiments on multiple days, with additional technical replicates within each biological replicate as appropriate. The experimental unit is indicated in each figure legend (e.g., molecules, trajectories, cells, or mice), and exact sample sizes (n) are reported there. For MINFLUX structural imaging, each condition includes measurements from ≥3 cells across ≥3 independent experiments/replicates. Because several primary readouts are distribution-based (e.g., inter-blade distance distributions), data acquisition was continued in some conditions to increase the precision of the estimated distribution (i.e., to better capture the full range of observed conformations), without performing interim significance testing and without excluding data based on outcomes. For electrophysiology, recordings were collected across independent transfections and days, with untransfected controls measured on each experimental day and manipulations paired with matched controls. All key findings were reproduced in independent experiments as noted in the figure legends.
Data exclusions	For electrophysiology, cells with high access resistance (> 20 MΩ) or low seal resistance (< 1 GΩ) were excluded from data analysis. Cells that changed their morphology during repetitive poke stimulation were also excluded from data analysis. Cells that developed membrane blebs during hypoosmotic challenge were excluded from data analysis. DRG neurons that exhibited a sudden increase in current of several hundred pA or several nA after the application of hypotonic solution, without recovering upon reintroduction to the iso-osmotic solution, were excluded from data analysis due to the potential compromise of the gigaseal stability or plasma membrane integrity during cell swelling.
Replication	At least three biological and experimental replicates were performed for each experiment, and experiments were performed over at least two separate days. All attempts at replication were successful.

Randomization	This study did not allocate experimental units to groups, and so no randomization was required for any experiment reported.
Blinding	For the siRNA knockdown experiments in Fig. 3c, the experimenter was blinded to the condition during the recordings. All other electrophysiology experiments were conducted as previously published without blinding. For all MINFLUX experiments, data were analyzed using automated analysis algorithms with the same settings, so blinding was not necessary. For all other experiments, blinding was not necessary since there were no comparisons.

Reporting for specific materials, systems and methods

We require information from authors about some types of materials, experimental systems and methods used in many studies. Here, indicate whether each material, system or method listed is relevant to your study. If you are not sure if a list item applies to your research, read the appropriate section before selecting a response.

Materials & experimental systems

n/a	Involved in the study	
☐	☒	Antibodies
☐	☒	Eukaryotic cell lines
☒	☐	Palaeontology and archaeology
☐	☒	Animals and other organisms
☒	☐	Clinical data
☒	☐	Dual use research of concern
☒	☐	Plants

Methods

n/a	Involved in the study	
☒	☐	ChIP-seq
☒	☐	Flow cytometry
☒	☐	MRI-based neuroimaging

Antibodies

Antibodies used	Primary antibodies: guinea pig anti-FLAG (a gift of David Ginty, described in doi: 10.1016/j.neuron.2023.08.023), rabbit anti-FLNB (Thermo Fisher #PA5-52098), and chicken anti-NFH (Abcam ab4680).<br>Secondary antibodies: goat anti-guinea pig Alexa Fluor 594 (Life Technologies A11076), goat anti-rabbit Alexa Fluor 647 (Life Technologies A21245), goat anti-chicken Alexa Fluor 488 (Life Technologies A32931), goat anti-guinea pig STAR RED (Abberior #STRED-1006), goat anti-rabbit STAR ORANGE (Abberior #STORANGE-1002).
Validation	All antibodies are previously published for use in mouse tissue. For commercially available antibodies, citations are available on the manufacturer's website. For the guinea pig anti-FLAG, use on mouse tissue is described in doi: 10.1016/j.neuron.2023.08.023 and https://doi.org/10.7554/eLife.10874. We always performed no-primary control experiments to validate lack of staining with secondary antibodies.<br>The primary antibodies were validated as follows:<br>Guinea pig anti-FLAG (from https://doi.org/10.7554/eLife.10874): " In CbfbFlag mice, the Flag antibody allows for specific detection of endogenous Flag-CBFβ, which appears to be expressed in nearly all DRG neurons, at varying levels (Figure 4A, B)." The authors performed staining of CbfbFlag on CbfbFlag mice and WT controls and observed specific staining.<br>Rabbit anti-FLNB (from manufacturer's website): "Relative expression in western blot: Antibody specificity was demonstrated by detection of known differential basal expression of the target across tissue/cell models. Expression of Filamin B was observed specifically in A-549 cells and in HEK293 cells using anti-Filamin B Polyclonal Antibody (Product # PA5-52098) in western blot. The relative expression levels of Filamin B within each cell line is shown using RNA-Seq."Relative expression in different tissues in IHC: Detection of differential expression levels of Filamin B demonstrates antibody specificity. Immunohistochemical analysis of Filamin B using anti-Filamin B Polyclonal Antibody (Product # PA5-52098), shows significant staining of Filamin B in human prostate and shows minimal or weak staining in human skeletal muscle tissues. The relative expression levels of Filamin B within each tissue is shown using RNA-Seq."<br>Chicken anti-NFH (from manufacturer's website): "Anti-Neurofilament heavy polypeptide antibody (ab4680) is a Chicken Polyclonal antibody and is validated for use in ICC, IHC-FrFl, WB. Anti-Neurofilament heavy polypeptide antibody (ab4680) has been cited over 135 times in peer reviewed journals and is trusted by the scientific community. Abcams high quality validation processes ensure Anti-Neurofilament heavy polypeptide antibody (ab4680) has high sensitivity and specificity. Anti-Neurofilament heavy polypeptide antibody (ab4680) has 12 independent reviews from customers." Example citation using this antibody in our laboratory: https://doi.org/10.1038/s41586-022-04860-5.

Eukaryotic cell lines

Policy information about cell lines and Sex and Gender in Research

Cell line source(s)	Swell1-knockout cells were of the Freestyle HEK293-F cell line, originally obtained from ThermoFisher Scientific, and modified as described in Kefauver, et al. 2018. PtK2 (NBL-5) cells were obtained from ATCC (ATCC CCL-56). Clonal Flnb knockout cells were custom made using CRISPR-Cas9 on the Swell1-knockout HEK293-F cell line background as described in the methods. Expi293 cells were obtained from ThermoFisher Scientific.
Authentication	Commercially available cell lines were authenticated by the supplier. Expi293 cells (from ThermoFisher Scientific) were authenticated for post-thaw viability, mycoplasma testing by PCR, and sterility testing. PtK2 cells (from ATCC) were autheticated by morphological analysis, species verification by isoenzymology, STR profiling, and mycoplasma testing.

Knockout of the genes encoding Swell1 (LRRC8A, LRRC8B, LRRC8D, and LRRC8E) in the Swell1-KO cells were verified previously in Kefauver, et al. 2018. Successful knock-out of Swell1 genes was determined by PCR genotyping and Sanger sequencing targeted regions for frameshift mutations and verified by mass spectrometry analysis. Knockout of Flnb in clonal Flnb + Swell1-KO knockout cells was determined by PCR genotyping and Sanger sequencing targeted regions for frameshift mutations.

Mycoplasma contamination

All cell lines tested negative for mycoplasma contamination using the MycoAlert® Mycoplasma Detection Kit (Lonza).

Commonly misidentified lines
(See ICLAC register)

None

Animals and other research organisms

Policy information about studies involving animals; ARRIVE guidelines recommended for reporting animal research, and Sex and Gender in Research

Laboratory animals

Mice were kept in standard housing with a 12 hour light/dark cycle, with lights on from 6 AM to 6 PM. Room temperature was kept around 22C, and humidity between 30-80% (not controlled). Mice were kept on pelleted paper bedding and provided with paper square nestlets and PVC pipe enrichment. Mice were given ad libitum access to food and water. Mouse ages ranged from 2.5 weeks to 4 months of age for IHC and smFISH experiments. WT mice used for electrophysiology and smFISH experiments were C57BL6/J background (Jackson Laboratories Strain #:000664). Piezo2-smFP-FLAG mice used for IHC experiments were a gift of David Ginty (Piezo2em1.1Ddg/J, CD-1 genetic background, Jackson Laboratories Strain #039935). PCR genotyping from tail snip DNA samples was performed in house using guidelines from Jackson Laboratory and/or through Transnetyx. All mice except for C57BL/6J mice were given metal identification tags (National Band & Tag, 1005-1) on the right ear at an age of 18-30 days old. Upon weaning between 21-30 days of age, mice were co-housed in groups of 2-5 littermates of the same sex.

Wild animals

This study did not involve wild animals.

Reporting on sex

Sex was not considered in study design.

Field-collected samples

This study did not involve field-collected samples.

Ethics oversight

All experiments were approved by the Scripps Research Animal Care and Use Committee under protocol # 08-0136. All experiments were performed under the policies and recommendations of the International Association for the Study of Pain and approved by the Scripps Research Animal Care and Use Committee.

Note that full information on the approval of the study protocol must also be provided in the manuscript.

Plants

Seed stocks

Report on the source of all seed stocks or other plant material used. If applicable, state the seed stock centre and catalogue number. If plant specimens were collected from the field, describe the collection location, date and sampling procedures.

Novel plant genotypes

Describe the methods by which all novel plant genotypes were produced. This includes those generated by transgenic approaches, gene editing, chemical/radiation-based mutagenesis and hybridization. For transgenic lines, describe the transformation method, the number of independent lines analyzed and the generation upon which experiments were performed. For gene-edited lines, describe the editor used, the endogenous sequence targeted for editing, the targeting guide RNA sequence (if applicable) and how the editor was applied.

Authentication

Describe any authentication procedures for each seed stock used or novel genotype generated. Describe any experiments used to assess the effect of a mutation and, where applicable, how potential secondary effects (e.g. second site T-DNA insertions, mosiacism, off-target gene editing) were examined.
