# Supplementary material for: Author Correction: The molecular basis of force selectivity by PIEZO2
Source: Nature. 2026 Sep 10;657(8133):E22. doi: 10.1038/s41586-026-11079-1 (PMC13600929; doi:10.1038/s41586-026-11079-1)
Supplement: Supplementary file 1 [file 41586_2026_11079_MOESM1_ESM.pdf]

nature portfolio
https://doi.org/10.1038/s41586-026-11079-1

Supplementary information

Author Correction: The molecular basis of force selectivity by PIEZO2

In the format provided by the
authors and unedited

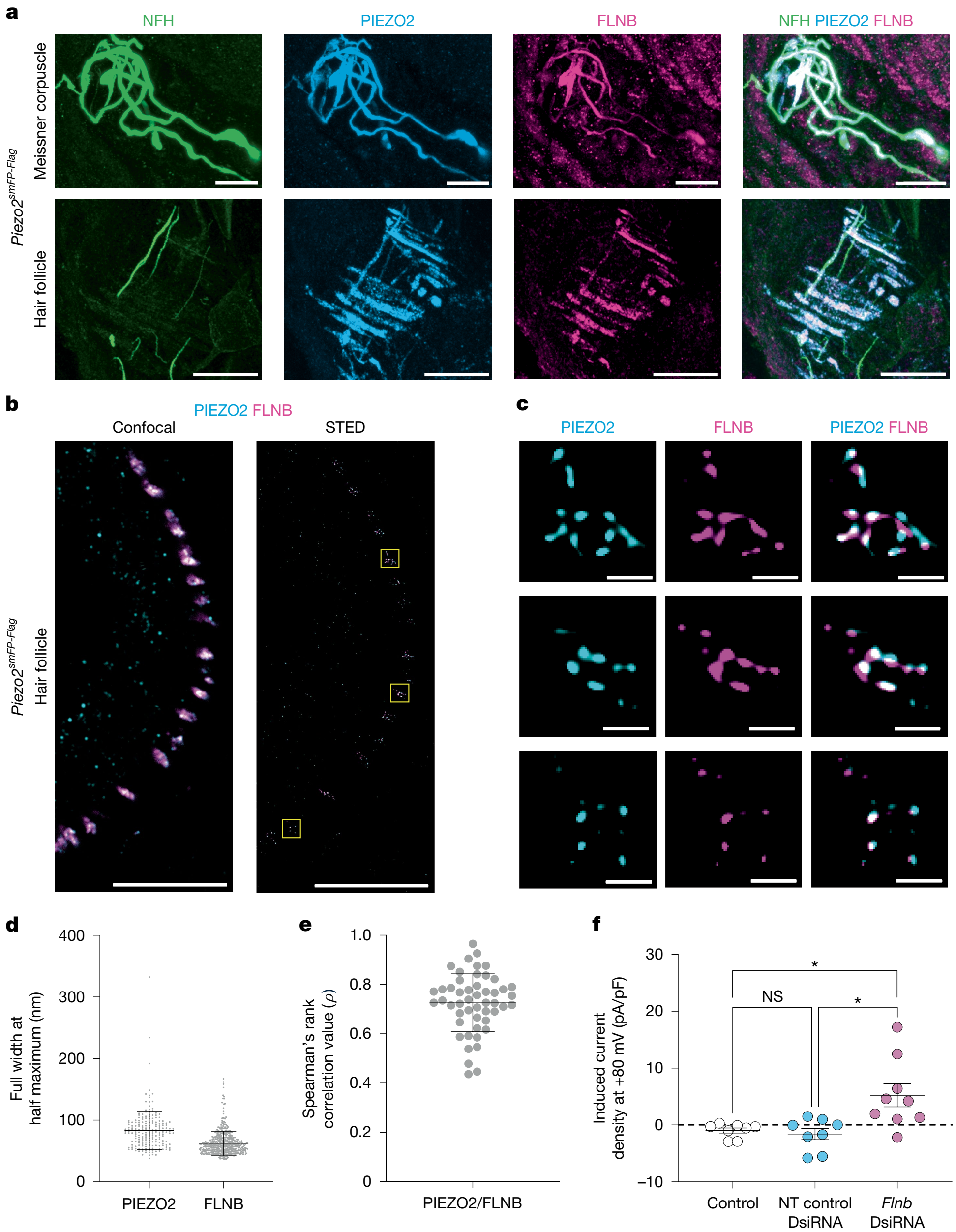

Fig. 4 | FLNB and PIEZO2 in somatosensory neurons. a, Representative images of sectioned skin from *Piezo2*$^{smFP\text{-}Flag}$ mice that contain end-organs formed by *Ntrk2*$^{+}$ and/or *Ntrk3*$^{+}$ LTMRs: lanceolate endings around hair follicles from back skin (top, five sections from two mice) and Meissner corpuscles from glabrous skin of paw digits (bottom, six sections from one mouse). Each section was co-stained with antibodies against NFH, PIEZO2–Flag and FLNB. Scale bars, 10 µm. **b**, Representative super-resolution STED microscopy image of PIEZO2–Flag and FLNB immunostaining in lanceolate endings showing co-localization of single puncta (six sections from two mice). Scale bars, 10 µm. **c**, Isolated single lanceolates from the yellow boxes in **b** (representative images of six sections from two mice). Scale bars, 500 nm. **d**, FWHM resolution of all identified peaks. Mean ± s.d. FWHM: anti-PIEZO2–Flag = 83 ± 31 nm, anti-FLNB = 62 ± 19 nm. *n* = 201(PIEZO2) and *n* = 469 (FLNB) puncta. **e**, Quantification of colocalization using Spearman's rank correlation value $\rho = 0.72 \pm 0.12$ (mean ± s.d.). *n* = 50 lanceolates from 5 follicles and 2 mice. **f**, The amplitude of stretch-induced currents in dissociated large diameter (≥70 µm) DRG neurons without any treatment (control, *n* = 8 cells), nucleofected with non-targeting DsiRNA (*n* = 8 cells) or nucleofected with DsiRNAs targeting *Flnb* (*n* = 9 cells). *n* = 3 mice for each condition. Two-sided Kruskal–Wallis test with Dunn's multiple-comparison test; *P* = 0.9999 (control versus non-targeting DsiRNA), **P* = 0.0105 (control versus *Flnb* DsiRNA), **P* = 0.0201 (non-targeting DsiRNA versus *Flnb* DsiRNA).
